# The Brazilian Society for Cardiovascular Surgery (SBCCV) and Brazilian Society for Extracorporeal Circulation (SBCEC) Standards and Guidelines for Perfusion Practice

**DOI:** 10.21470/1678-9741-2018-0347

**Published:** 2019

**Authors:** Luiz Fernando Caneo, Gregory Matte, Robert Groom, Rodolfo A. Neirotti, Paulo Manuel Pêgo-Fernandes, Juan Alberto C. Mejia, Fernando Augusto Marinho dos Santos Figueira, Élio Barreto de Carvalho Filho, Fábio Murilo da Costa, Sintya Tertuliano Chalegre, Renato Abdala Karam Kalil, Rui M. S. Almeida, DECAM/SBCCV, SBCECV

**Affiliations:** 1 Cardiovascular Surgery Division, Instituto do Coração, Hospital das Clínicas da Faculdade de Medicina da Universidade de São Paulo (InCor-HCFMUSP), São Paulo, SP, Brazil.; 2 Department of Cardiac Surgery, Boston Children's Hospital, Boston, MA, USA.; 3 Maine Medical Partners - Cardiothoracic Surgery, Portland, USA.; 4 Clinical Professor of Surgery and Pediatrics, Emeritus Michigan State University, MI, USA.; 5 Thoracic Surgery Division of the Instituto do Coração, Hospital das Clínicas da Faculdade de Medicina da Universidade de São Paulo (InCor-HCFMUSP), São Paulo, SP, Brazil.; 6 Unidade de Transplante e Insuficiência Cardíaca do Hospital de Messejana Dr. Carlos Alberto Studart Gomes, Fortaleza, CE, Brazil.; 7 Instituto de Medicina Integral Professor Fernando Figueira (IMIP), Recife, PE, Brazil.; 8 Universidade Estadual de Campinas (Unicamp), Campinas, SP, Brazil.; 9 Pronto-Socorro Cardiológico de Pernambuco, (PROCAPE), Recife, PE, Brazil.; 10 Universidade de Pernambuco (UPE), Recife, PE, Brazil.; 11 Instituto de Cardiologia do Rio Grande do Sul - Fundação Universitária de Cardiologia, Porto Alegre, RS, Brazil.; 12 Universidade Estadual do Oeste do Paraná (UNIOESTE), Cascavel, PR, Brazil.; 13 Departamento de Assistência Circulatória Mecânica (DECAM) da Sociedade Brasileira de Cirurgia Cardiovascular (SBCCV), São Paulo, SP, Brazil.; 14 Sociedade Brasileira de Circulação Extracorpórea (SBCEC), São Paulo, SP, Brazil.

A primary role for clinical medicine societies is to develop standards and guidelines for practice as an instrument to promote safe and effective patient care. The Brazilian Society for Cardiovascular Surgery (SBCCV) represented by its Department for Mechanical Circulatory Assistance (DECAM) and the the Brazilian Society for Extracorporeal Circulation (SBCEC) conducted a careful critical review of current clinical perfusion practices in Brazil. In addition, a literature review focused on patient safety and surgical outcomes in cardiac surgery was performed. This is the first joint initiative of these two societies (SBCCV/SBCEC) to provide a framework for safe and effective clinical perfusion practice for our cardiac surgery patients. The purpose of this pioneering work was to develop guidelines for the perfusion profession and for those involved in cardiopulmonary bypass (CPB) technology in our country. Both the SBCCV and the SBCEC recommend that institutions and clinical teams adopt the standards and guidelines outlined in this text. The standards and guidelines we recommend are based on those published by the published American Society for Extracorporeal Technology (AmSECT) with a phased adoption recommendation set as an achievable goal. Further, we recommend that cardiac surgery programs develop institution-specific protocols to support the clinical use of these guidelines.

## The Pioneering Era of Cardiac Surgery

Open heart surgery has developed considerably over the past several decades including numerous pioneering efforts in Brazil regarding biomedical engineering and circulatory support^[1]^. Pioneer surgeons, such as John Kirklin, Francis Fontan, Euryclides Zerbini, Adib Jatene, and Denton Cooley were part of our lives and we were able to study their papers, witness their presentations and participate in professional discussions. They are passing away one after another but their work, techniques, experience and wisdom stays with us as their legacy. The impact of their methods profoundly changed the lives of our patients with congenital heart defects, giving them the chance of enjoying a better quality of life. Now, the pioneering era of cardiac surgery has essentially ended in Brazil.

Congenital cardiac surgery is markedly changing and surgeon-centered outcomes are being replaced by team-based efforts with new paradigms requiring an adaptive work environment in institutions where cardiac surgery is performed.

As William Norwood aptly put in his paper, *Our Roots, Our Future* ^[1,2]^, "Institutions are not what they are by historical prerogative: the people walking the halls are responsible for maintaining the legacy and creating new vistas." That being said, we need to continue the initial work of our pioneers and press on upgrading their achievements to ever higher standards. The era we have now entered is no longer about quantity, it is about achieving excellent whole-patient quality outcomes including optimized neurologic outcomes. We must dig deep into issues that impact the quality of outcomes, teamwork and overall transparency in our respective professions.

## Reviewing Perfusion Practice: Time to Stop Living in the Past

Brazil has a strong history of innovation that extends back to the earliest days of cardiac surgery when our centers pioneered advances in heart-lung machines (HLMs), cardiac valves, conduit implants, and surgical techniques. Brazil started to produce their own HLMs in 1959 and indeed used one of them to perform the first heart transplantation in South America. These innovations highlighted the teamwork primarily of surgeons and biomedical engineers. This was natural since surgeons and other physicians were the first 'perfusionists'. Additionally, perfusion products, including a series of oxygenators, were developed and manufactured domestically. While we fondly remember these great achievements, we also need to focus on the future. Unfortunately, there are still people living in the past and not adapting to evolving cardiac surgery and perfusion practices. We continue to blame our economic burden for the stagnation of our practice while paying little to no attention to the need for cultural change in the operating room.

Furthermore, clinical perfusion has not been recognized by the government as a distinctive profession until quite recently. Currently, only five professional councils recognize Perfusion as a specialty for their undergraduates: Biology, Biomedicine, Nursing, Pharmacy, and Physiotherapy. These professions do not have a standardized perfusion-specific curriculum. Consequently, perfusionist education and training is heterogenous. Furthermore, it is still the case in Brazil that perfusionists must follow the instructions of surgeons and anesthesiologists. In fact, the conduct of perfusion is only considered a medical act once the perfusion record is signed by the surgeon. This practice risks perfusionists not taking full ownership for the conduct of CPB and that raises serious safety concerns since the surgeon and anesthesiologist have much to attend to during cardiac surgery and the perfusionist is the individual who can best manage extracorporeal support with all of its nuances. These facts support the outdated paradigm whereby perfusionists are essentially asked to follow the instructions of surgeons and anesthesiologists during CPB instead of working collaboratively within a famework of well-developed perfusion practice guidelines. Currently, the SBCEC and the SBCCV are in discussion with the Federal Councils regarding ways for this activity to be uniformly recognized by the Professions and subsequently legalized with a federal law regulating perfusion activities. Brazilian perfusionists must have the education, tools and authority to perform their job and to become active and respected members of the multidisciplinary cardiac surgery team. Several limitations currently exist which impair the advancement of perfusion practice, including educational gaps, a lack of case ownership, and a lack of tools to assess the adequacy of perfusion in real time during surgery. This is a vicious cycle which impacts outcomes and patient safety.

On a positive note, it is important to highlight the progress made by the Brazilian Society of Extracorporeal Circulation. Supported and stimulated by their society, a significant number of Brazilian perfusionists have had the opportunity to attend symposium-based perfusion related courses, exchange experiences with more advanced international programs and to discuss current techniques of extracorporeal circulation with local perfusionists and those from abroad.

In more developed countries, perfusionists have the freedom to choose perfusion products according their performance, their patient population's needs, and the information available in the literature. Each component is selected via an independent decision with the ideal components used to build the circuit. Alternatively, in Brazilian perfusion practice, it is difficult to be objective since product decisions are almost exclusively based on price and subjective preferences due to the lack of scientific publications comparing Brazilian perfusion products with those available in other markets. In our country, oxygenator manufacturers typically provide complimentary HLMs with an agreement that their oxygenator can only be guaranteed on their HLM. There is an obvious conflict of interest with such an agreement. This implied agreement has no scientific basis and, to our knowledge, is not practiced elsewhere which speaks to the need for change in Brazilian cardiac surgery. Again, our culture needs to adapt to end such practices for the benefit of our patients. This is even more of a concern when one notes that the majority of HLMs made and used in Brazil do not have servoregulating safety devices incorporated for arterial flow, cardioplegia delivery, level sensing, and bubble detectors. Servoregulation for HLM functions is not enough. Perfusionists must also be trained to operate the devices. Standards for perfusion practice, including the use of safety devices, must be established and adhered to.

## Why are Clinical Perfusion Standards so Important?

The Gritten Report^[3]^ published by the University Hospitals of Bristol National Health Service (NHS) Foundation Trust of Great Britain described the death of a five-month-old infant undergoing complex cardiac surgery and was released May 25, 2005. The Root Cause Analysis (RCA) report was led by Mark Gritten, an independent and nationally known NHS senior professional. A police investigation and coroner's inquest labeled the case 'unlawful killing'. In English law, unlawful killing means that the killing was made without lawful excuse and in violation of criminal law including murder, manslaughter, and infanticide. The finding of unlawful killing must be beyond reasonable doubt; that is, the evidence must be overwhelmingly obvious that death would result from the act when all factors are taken into account. Otherwise, a verdict of accidental death or death by misadventure would apply. The death was the result of a calcium overdose by a perfusionist that caused irreversible brain damage and subsequent death the day after surgery. The hospital put safeguards into place immediately to minimize any similar incidents happening again. Also, the National Society of Perfusionists perhaps carried some responsibility for this incident because it does not appear to have disseminated other perfusion incidents between its members.

The report concluded that this was a unique but avoidable incident that resulted in an indictment not just to the perfusionist involved in the accident, but to all perfusionists and the perfusion profession as a whole in Great Britain. Had a similar incident happened in São Paulo or Rio or elsewhere in Brazil, would the SBCEC or SBCCV also be held responsible?

Perfusion practice during cardiovascular surgery is recognized in the international literature as a critical component to successful patient outcomes. Therefore, as medical societies, we have the responsibility to change our culture, our commercial practices, legislation, regulations and whatever else which involves our specialty which can improve patient outcomes^[4,5]^. The intent of our proposed standards and guidelines document is to provide a modern framework for the practice of cardiopulmonary bypass in Brazil that can maximize patient safety and outcomes.

The standards and guidelines document we developed for perfusion practice in Brazil is based on publications from AmSECT^[6,7]^. It focuses on the role of written institutional protocols to dictate clinical practice. We worked on four main subjects:

- empowerment of perfusion as profession with a focus on professional qualification and education standards
- standardization of perfusion practices
- mandatory safety devices
- importance of non-technical skills and patient centered team work

## Professional Constraints:

Although perfusion is considered a medical act, Perfusion as a profession is still not fully regulated in Brazil. Consequently, the legal responsibility for what happens at the pump is unclear. The surgeon's knowledge of what is actually happening on the pump at all times during an operation depends upon their communications with the perfusionist. The surgeon's signature on the perfusion record is a formality which does not ensure proper care during CPB. This practice jeopardizes the development of a new generation of perfusionists who should be taking ownership for their individual perfusion cases and, of course, introducing the necessary changes to modernize existing clinical practices. The Perfusionist must be responsible for the whole procedure of extracorporeal circulation and be an active member of the cardiac surgery team, as is the case with most enters abroad.

According to the SBCEC, perfusionists are expected to have:

- Dedication to the patient
- Full integration with the team in which they work
- Professional competence
- Personal ethical and professional conduct, as well as being zealous, affable, aware and observant.

Considering our context, the effort of publishing this document by the societies SBCCV and SBCEC should be considered as one of most important steps for the future of cardiopulmonary bypass practice in Brazil.

The "holy trinity" for the cardiac surgey patient- perfusionist, surgeon and anesthesist- is a critical issue for optimal outcomes in cardiac surgery. Therefore, publication in Brazil of the Standards and Guidelines for Perfusion Practice aims not only to improve CPB but also to improve overall surgical outcomes as an important quality improvment initiative.

## Perfusion and the Pediatric Cardiac Surgery:

In the early 1950s, the pioneers of congenital cardiac surgery, among them- Bigelow, Lewis, Kirklin, Gibbon and others- realized that the time available with hypothermia and inflow occlusion would not be sufficient to safely perform lenghty intracardiac operations and that an extracorporeal support system would be needed. In 1954, Lillehei introduced the technique of controlled cross-circulation, in which a patient's parent functioned as the extracorporeal pump and oxygenator- a system that put both the parent and the child at risk. Therefore, the development of mechanical cardiopulmonary bypass circuits in the late 1950s was an important step for the progress of congenital cardiac surgery. Since then, extracorporeal perfusion circuits have come a long way to the current low prime membrane oxygenators, the use of centrifugal pumps, vacuum-assisted venous drainage, electronic gas blenders, in-line oxygen analyzers and other important devices.

The array and complexity of the equipment, the perfusion techniques to manage a wide variety of patient's age and size along with the broad spectrum of surgical procedures are real challenges that require properly trained and knowledgeable perfusionists.

Because one size does not fit all, the need for a standalone Standards and Guidelines document to perform perfusion for congenital heart surgery is unquestionable and it will in many ways be unique as compared to the one used for the correction of acquired heart disease in adults.

Providing cardiopulmonary support for repair of congenital heart lesions has become a specialty standing on its own. This context should determine the strategies and processes to address these issues; the professionals, administrators, and professional societies should be engaged in planning, setting and articulating the goals of robust pediatric perfusion standards and guidelines to improve the outcomes in pediatric cardiac surgery.

The Brazilian Society for Cardiovascular Surgery (SBCCV) and the Brazilian Society for Extracorporeal Circulation (SBCEC) Standards and Guidelines for Perfusion Practice address perfusion in general. We believe that developing a specific Brazilian Pediatric Perfusion Standards and Guidelines document is essential and that it should be published in the near future to complement this document.

## Development of this Document

The Standards and Guidelines for Perfusion Practice will serve as a useful guide for Brazilian cardiac surgical teams to develop institution-specific protocols aimed at improving the reliability, safety, and effectiveness of cardiopulmonary bypass. We are aware that the development of a Standards and Guidelines for Perfusion document alone will not change patient care or outcomes. Safe, reliable, and effective care will be best served through the implementation of institutional protocols based on these standards. SBCCV/SBCEC's Standards and Guidelines for Perfusion Practice reflect the changing landscape for perfusion leading to the safe and optimal provision of cardiopulmonary bypass for our patients as well as a working team-based environment that is supportive of these policies.

We preferred to name this document "Standards and Guidelines for Perfusion Practice" because this terminology is contemporary and coincides with the language used by other professional medical societies, including AmSECT ^[8]^.

The SBCCV/SBCEC Standards and Guidelines for Perfusion Practice: 2018 is primarily based on a previous document developed by AmSECT, through its Perfusion Quality Committee. Initially, AmSECT developed a draft standard for perfusion entitled the "Essentials for Perfusion Practice, Clinical Function: Conduct of Extracorporeal Circulation," which was originally endorsed by the membership in 1993^[9]^, and then reviewed and revised on a number of occasions^[10-12]^. In 2011, the AmSECT Board of Directors (BOD) asked the International Consortium for Evidence-Based Perfusion (ICEBP) subcommittee to review and update the "Essentials and Guidelines" document. The ICEBP conducted a careful review and critique of the document as well as its relevance and purpose, given the focus on patient safety and surgical outcomes. This initiative resulted in a revised joint document entitled, the Report from AmSECT's, International Consortium for Evidence-Based Perfusion American Society of ExtraCorporeal Technology Standards and Guidelines for Perfusion Practice: 2013^[13]^. It was developed as an outgrowth of marrying evidence-based practices from the literature with an understanding of the context in which care is currently provided. Quite notably at the same time, the Minimum Standards for Perfusion Practice in Brazil document was developed as an outgrowth of ongoing collaboration with the International Quality Improvement Collaborative for Congenital Heart Surgery (IQIC) which is managed from Boston Children's Hospital and overseen by an international steering committee. Adoption of the Minimum Standards for Perfusion Practice in Brazil document will empower perfusionists to effect change at their institution by working towards practice standards endorsed by their national organizations including minimum safety devices for all cardiopulmonary bypass cases, monitoring devices to help assess the adequacy of perfusion, and promotion of a team-based appoach for the care of cardiac surgical patients. Our vision to improve perfusion practice, and thus patient outcomes, is for the minimum standards to be adopted as soon as possible by Brazilian cardiac surgery teams with the comprehensive list of AmSECT standards phased in as soon as practial given the constraints discussed previously.

Following translation to Portuguese and critical review by colleagues, this final document was presented to the SBCCV and SBCEC for their steering commitiee aproval. A majority of the members of the steering commities of both societies voted to accept this document as an official position for the Standards and Guidelines for Perfusion Practice in Brazil. Both documents are included in this manuscript. The SBCCV and SBCEC endorse this comprehensive report and strongly recommend implementation.

## Minimum Standards for Perfusion Practice in Brazil:

Seven standards were identified as the minimum recomendation for perfusion practice. The SBCCV and SBCEC considers these seven standards as mandatory for all cardiac surgical centers (Appendix 1).

## SBCCV/SBCEC Comprehensive Standards and Guidelines for Perfusion Practice in Brazil:

The Perfusion Standards listed in Appendix 2 have been modified and adapted to the Brazilian regulatoy agencies' policies and recommendations, by taking The American Society of ExtraCorporeal Technology (AmSECT) Standards and Guidelines as a model^[7]^ and translated to Portuguese. The final document consists of 15 areas of practice including 50 Standards and 38 Guidelines (Appendix 1) with the first standard focusing on the development of institutional protocols to support their implementation and use. Each institution must commit to working towards implementing all standards for patients undergoing cardiovascular surgery.

## Terminology

The SBCCV and SBCEC would like to point out that cardiac surgery clinicians must understand the terminology used in this report. The meanings of these words, as described in the AmSECT publications, are listed below in order to facilitate understanding and adoption of the Standards and Guidelines^[7]^:

**Standards:** practices, technology, and/or conduct of care that institutions shall meet to fulfill the minimum requirements for cardiopulmonary bypass

**Guidelines:** recommendation that should be considered and may assist in the development and implementation of protocols

**Protocols:** an institution-specific written document, derived from professional standards and guidelines, which contains decision and treatment algorithms

In this document, the word **shall** is used to indicate a **mandatory** requirement

In this document, the word **should** is used to indicate a **recommendation**

In this document, the term **surgical care team** is used to indicate the components of the system: surgeon, anesthesiologist, perfusionist, nurse, and technicians

## CONCLUSION

The SBCCV and SBCEC both recognize the vital need for cultural and clinical changes in the application of cardiopulmonary bypass in Brazil. Cardiac surgery centers must adopt the Minimum Standards For Perfusion Practice in Brazil as soon as possible and work towards adopting the Comprehensive Standards and Guidelines for Perfusion Practice in Brazil moving forward. Ultimately, a team-based approach utilizing nationally endorsed standards will help ensure safe and optimal cardiopulmonary bypass for all our patients and improve outcomes for the complex population we serve.

## Appendix 1

**Minimum Standards for Perfusion Practice in Brazil**

**Minimum Standard 1:** Perfusion practice must be guided by a written set of policies developed within the institution and approved by physician leadership. (See also Comprehensive Standard 1).

**Minimum Standard 2:** Each perfusionist must be adequately trained through a defined education process. Staff must participate in annual continuing education activities and institutional based quality improvement programs. (See also Comprehensive Standards 2 and 13).

**Minimum Standard 3:** The care team must discuss the bypass plan before incision; anticoagulation plan and target ACT values, pump flows, hematocrit management, target temperature, myocardial protection plan, blood gas management, blood pressure goals, etc. Closed-loop communication must be used during the procedure. The care team must have real-time multidisciplinary discussion regarding all concerns during bypass (*i.e.*, blood pressure too low, poor venous drainage, falling NIRS, need for blood product transfusion, etc.). (See also Comprehensive Standards 1, 3, 5, 8 and 12).

**Minimum Standard 4:** Perfusion equipment must be maintained by qualified personnel. An appropriately sized selection of equipment and standardized disposables must be used for each patient with back-up equipment available. Back-up supplies of cannulae and connectors, etc., must be located next to the primary perfusionist in the OR. The bypass circuit must be set-up on the heart-lung machine before the patient arrives in the operating room. (See also Comprehensive Standards 6 and 14).

**Minimum Standard 5:** The perfusion record must include sufficient timed data to reconstruct a bypass run, include the prebypass checklist and list the products used for the case. The perfusion record must be part of part of the patient's medical record. (See also Comprehensive Standards 3,4, 5, 7, 8, 9 and 12).

**Minimum Standard 6:** The follow monitoring and safety devices must be used for all patients; patient and circuit temperature probes, reservoir level sensor, bypass system and cardioplegia temperature and pressure, an arterial line filter, flow probe, one-way valve on the vent line, back-up oxygen supply, SvO2 monitoring and a hand crank. The following items should be considered for every case: NIRS monitoring and bubble detection. Servoregulation must be utilized where available. Blood gases must be verified on a defined schedule. (See also Comprehensive Standards 6, 7, 10 and 11).

**Minimum Standard 7:** The perfusion team must have adequate storage space near the operating theater for back-up and emergency supplies. A comfortable chair which allows for close monitoring of the perfusion circuit should be available to the perfusoinist during bypass. (See also Comprehensive Standard 14).

## Appendix 1

**Padrões Mínimos para a Prática de Perfusão no Brasil**

**Minimum Standard 1:** As práticas de perfusão devem ser orientadas por um conjunto de políticas desenvolvidas dentro da instituição, disponíveis na forma escrita e aprovadas pelo)a) chefe da equipe cirúrgica (para mais detalhes, veja Comprehensive Standard 1).

**Minimum Standard 2:** Cada perfusionista deve ser treinado adequadamente em um processo de formação definido. Toda a equipe de perfusionistas deve participar das atividades anuais de educação continuada e dos programas de melhoria de qualidade da instituição (para mais detalhes, veja Comprehensive Standards 2 e 13).

**Minimum Standard 3:** A equipe cirúrgica deve discutir o plano de perfusão antes da incisão na pele, assim como anticoagulação e valores de TCA alvo, fluxos de perfusão, hematócrito alvo e política transfusional, temperatura alvo, estratégia de proteção miocárdica e manuseio da pressão arterial. A comunicação em alça fechada (*closed loop*) deve ser usada durante todo o procedimento. A equipe deve promover uma discussão multidisciplinar em tempo real sobre todas as preocupações durante a circulação extracorpórea (ou seja, pressão arterial muito baixa, dificuldade com a drenagem venosa, queda dos valores da oximetria cerebral não invasiva - near-infrared spectroscopy - NIRS, necessidade de hemoderivados, dentre outras) (para mais detalhes, veja Comprehensive Standards 1, 3, 5, 8 e 12).

**Minimum Standard 4:** A manutenção do equipamento de perfusão deve ser realizada por pessoal qualificado. A escolha adequada dos equipamentos necessários e dos descartáveis padronizados em protocolo institucional deve ser realizada para cada paciente, assim como um equipamento de reserva deve estar disponível para a perfusão. Os suprimentos e componentes descartáveis do circuito (cânulas e conectores etc.) em duplicidade devem estar localizados o mais próximo possível do perfusionista responsável pelo caso, preferencialmente na sala de cirurgia. O circuito de circulação extracorpórea (CEC) deve ser montado na máquina de CEC antes que o paciente chegue à sala de operação (para mais detalhes, veja Comprehensive Standards 6 e 14).

**Minimum Standard 5:** A ficha (registro) de perfusão deve incluir dados suficientes para reconstruir a perfusão por inteiro, incluindo um checklist pré-CEC e contendo todas as drogas e descartáveis utilizados para a condução do caso. A ficha de perfusão deve ser parte do prontuário médico do paciente e deve ser anexada a ele. Uma cópia deve ser mantida com a equipe de perfusão (para mais detalhes, veja Comprehensive Standards 3, 4, 5, 7, 8, 9 e 12).

**Minimum Standard 6:** Os seguintes dispositivos de monitoração e segurança devem ser utilizados para todos os pacientes: sensores de temperatura no paciente, no circuito venoso e arterial e na cardioplegia; sensor de nível de reservatório; sensor de bolhas; sistema de pressão na linha arterial e na linha de infusão da cardioplegia; filtro de linha arterial; fluxômetro na linha arterial; válvula unidirecional na linha aspiração da aorta/átrio esquerdo; cilindro de oxigênio de reserva; equipamento de monitoração da SvO2; monitor de oximetria cerebral contínua (NIRS); detector de bolhas e "*hand cranck*" para manuseio manual da bomba arterial. A servorregulação deve ser utilizada sempre que disponível. Os gases no sangue devem ser verificados em uma rotina predefinida em protocolo (para mais detalhes, veja Comprehensive Standards 6, 7, 10 e 11).

**Minimum Standard 7:** A equipe de perfusão deve ter espaço de armazenamento adequado perto da sala de cirurgia para suprimentos de emergência. Uma cadeira confortável que permita um monitoramento próximo do circuito de perfusão deve estar disponível durante a perfusão (para mais detalhes, veja Comprehensive Standard 14).

## Appendix 2

**SBCCV/SBCEC Comprehensive Standards and Guidelines for Perfusion Practice in Brazil***

**Standard 1: Development of Institutionally based Protocols**

**Standard 1.1:** As a mechanism for applying each standard to clinical practice, an institution or service provider shall develop and implement an operating procedure (protocol) for each of the standards.

**Standard 1.2:** The protocol shall be:

- Approved by the Chairman of Cardiac Surgery, or his or her designee, Director of Perfusion or equivalent, and other relevant clinical governance committees if available.
- Reviewed and revised annually or more frequently when deemed necessary.

**Guideline 1.1:** Deviation from protocol may be at the discretion of the Surgical Care Team and should be documented in the perfusion record.

**Standard 2: Qualification, Competency, and Support Staff**

**Standard 2.1:** Extracorporeal circulation should only be performed by a professional trained among the professions recognized by SBCEC and SBCCV as competent to carry out the procedure; have a post-graduate degree recognized by the Brazilian Ministry of Education (MEC) with minimum workload described in article 12 of the Brazilian Standards for the Exercise of the Specialty of Perfusionist in Extracorporeal Circulation or SBCEC Specialist Degree^[1]^, validated by this society; or professionals who fall within the sole paragraph of Article 2 of the aforementioned Standard.

**Standard 2.2:** Perfusionist competency shall be assessed annually to evaluate compliance with departmental protocols.

**Standard 2.3:** The perfusionist shall attend, participate, and engage in perfusion-related continuing education courses on an annual basis.

**Standard 2.4:** Support staff shall be available on-site to assist the primary perfusionist during CPB procedures.

**Guideline 2.1:** An individual graduating from an accredited perfusion education program should complete all requirements for the Sociedade Brasileira de Circulação Extracorpórea (SBCEC) certification.

**Guideline 2.2:** A standardized process should be developed and followed to identify, orient, and educate support staff to ensure they have general knowledge of the duties performed by the perfusionist, flow of the operation, and location of primary and ancillary items required during CPB. Support staff may include a perfusionist, nursing, technical, or nontechnical staff.

**Guideline 2.3:** A standardized process to educate, train, and to evaluate annually perfusion staff should be developed and followed.

**Guideline 2.4:** It is recommended any perfusion procedure be conducted by two perfusionists, ensuring a better procedural safety.

**Guideline 2.5:** Use of Personal Protective Equipment: The perfusionist should use Personal Protective Equipment (PPE), such as masks, goggles and procedure gloves while conducting CPB. Gloves should be changed after drawing a blood sample, blood products administrtion and manipulation (blood bag) or whenever exposed to a blood splash^[3]^.

**Standard 3: Perfusion Record**

**Standard 3.1:** The perfusion record (written and/or electronic) for each CPB procedure shall be included as part of the patient's permanent medical record. The perfusion record shall be maintained and stored according to institution policy for retaining patient medical records.

**Standard 3.2:** The record shall include:

- Patient information including demographics and preoperative risk factors (Appendix A)
- Information sufficient to accurately describe the procedure, personnel, and equipment (Appendix B).
- Patient physiological parameters documented at a frequency determined by institutional protocol (Appendix C).
- Blood gas and anticoagulation monitoring results (Appendix D).
- Signature of the perfusionist (and all relief perfusionists) performing the procedure.

**Guideline 3.1:** There should be perfusion (writing and/or electronic) record for each performed CPB procedure. The perfusion record must include a free text space for recording comments, including verbal orders given by the medical staff, including verbal orders given by the supervising physician.

**Guideline 3.2:** The perfusion record should include the signatures of the physician(s) providing oversight for the CPB procedure.

**Guideline 3.3:** Raw data (*e.g*., blood flow, pressure, and temperature values) contained in electronic perfusion databases should be stored for a time period in accordance with your institution's policy for retaining electronic patient medical records.

**Standard 4: Checklist**

**Standard 4.1:** The perfusionist shall use a checklist for each CPB procedure^[4]^.

**Standard 4.2:** Checklists shall be included as part of the patient's permanent medical record.

**Guideline 4.1:** The perfusionist should use checklists in a read-verify manner where critical steps that should have been performed are confirmed^[4]^. Completion of the check-list should be performed by two people, one person being the primary perfusionist responsible for operation of the heart lung machine during the intraoperative period. In services where there is no availability of another professional perfusionist, a systematic routine of checking the items contained in the checklist should be adopted, in order to minimize the occurrence of adverse events.

**Guideline 4.2:** The perfusionist should use a checklist throughout the entire perioperative period (*e.g.*, setup, pre-bypass, initial onset of bypass, before cessation of bypass, postbypass, and/or any return to bypass).

**Guideline 4.3:** The perfusionist should use a checklist for other ancillary perfusion services (*e.g.*, cell salvage, intra-aortic balloon pump, extracorporeal membrane oxygenation).

**Standard 5: Communication**

**Standard 5.1:** A patient-specific management plan for the CPB procedure shall be prepared and communicated to the surgical team either during the preoperative briefing or before beginning the procedure^[5]^.

**Guideline 5.1:** The use of cellular telephone technology in the operating room should be avoided, since it is a distracting factor and predispose the patient to the risk of contamination by potentially infectious bacteria, compromising the quality and safety of the health assistance. When their use is unavoidable, the devices must be pre-sanitized according to the institution protocol for infection control^[6-8]^.

**Guideline 5.2:** Protocol-driven communication (*e.g*., closed-loop) should be used to acknowledge verbal commands, verify the content, and reduce ambiguity^[9-11]^.

**Guideline 5.3:** The primary perfusionist should participate in the postprocedure debrief with the surgical team.

**Standard 6: Safety Devices**

**Standard 6.1:** Pressure monitoring of the arterial line, cardioplegia delivery systems, and venous reservoir (when vacuum assisted venous drainage is used) shall be used during CPB procedures.

- The pressure monitor shall be either servoregulated to control the arterial/cardioplegia pump or to allow interruption to the arterial/cardioplegia flow.
- The pressure monitor shall include an audible and visual alarm.

**Standard 6.2:** A bubble detector shall be used during CPB procedures.

- The gross/macrobubble detector shall be used to control the arterial pump or to allow interruption of the arterial blood flow.
- The detector system shall include an audible and visual alarm and be positioned according to manufacturer instructions for use to enable timely identification and action.

**Standard 6.3:** A level sensor shall be used during CPB procedures using a (hard-shell) reservoir.

- The level sensor shall be either servo-regulated to control the arterial pump or to allow interruption of the arterial blood flow.
- The level sensor shall include an audible and visual alarm and be positioned according to the manufacturer's instructions to allow an appropriate reaction time and a safe operational volume.

**Standard 6.4:** Temperature monitoring of the arterial outflow from the oxygenator shall be used during CPB procedures.

- The temperature sensor shall include an audible and visual alarm to prevent high arterial outlet temperatures.

**Standard 6.5:** An arterial-line filter shall be used during CPB procedures.

**Standard 6.6:** A one-way valve in the vent line shall be used during CPB procedures.

**Standard 6.7:** A method for retrograde flow avoid- ance when using a centrifugal pump shall be used during CPB procedures.

- At least one method to prevent retrograde flow shall be used for systems using centrifugal pumps for systemic circulation. Examples of retrograde avoidance systems may include the following:
- One-way flow valves;
- Hard-stop detent controls to prevent accidental reduction in pump speed;
- Electronically activated arterial line clamps; or
- A low-speed visual and audible alarm.

**Standard 6.8:** An anesthetic gas scavenge line shall be used whenever inhalation agents are introduced into the circuit during CPB procedures.

**Standard 6.9:** Hand cranks shall be readily available during CPB procedures.

**Standard 6.10:** A back-up gas supply shall be available during CPB procedures.

**Standard 6.11:** A back-up battery supply for the CPB machine shall be available during CPB procedures.

**Guideline 6.1:** A ventilating gas oxygen analyzer should be used during CPB procedures.

**Guideline 6.2:** A level sensor should be used during CPB procedures using a soft-shell reservoir.

- The level sensor should be either servo-regulated to control the arterial pump or to allow interruption of the arterial blood flow.
- The level sensor should include an audible and visual alarm and be positioned according to manufacturer's instructions to allow an appropriate reaction time and a safe operational volume.
- The use of an air bubble detector distal to the outlet can be used as a surrogate level detector.

**Guideline 6.3:** The tubing pack should be provided by the manufacturer "pre-assembled", pre-connected and in a sterile tray separating the circuit that will be used in the surgical field of the one that will be mounted in the heart-lung machine (HLM), offering more safety to the perfusion itsef and granting faster circuit assembly.

**Standard 7: Monitoring (obs: to be performed in conjunction with Standard 3)**

**Standard 7.1:** Patient arterial blood pressure shall be monitored continually during CPB.

**Standard 7.2:** Arterial line pressure shall be monitored continually during CPB.

**Standard 7.3:** Arterial blood flow shall be monitored continually during CPB.

**Standard 7.4:** Cardioplegia dose, delivery method, line pressure (antegrade), coronary sinus pressure (retrograde), and ischemic intervals shall be monitored continually during CPB.

**Standard 7.5:** Patient and device temperatures shall be monitored continually during CPB.

- Patient (*e.g*., nasopharyngeal, rectal, bladder, esophageal).
- Heart-lung machine (arterial, venous and cardioplegia).
- Heater cooler (H_2_O temperature).

**Standard 7.6:** Blood gas analyses shall be monitored con- tinually or at regular intervals during CPB (Appendix D).

**Standard 7.7:** Hematocrit (or hemoglobin) shall be monitored continually during CPB.

**Standard 7.8:** Oxygen fraction and gas flow rates shall be monitored continually during CPB (Appendix D).

**Standard 7.9:** The percentage of venous line occlusion of the venous occluder shall be monitored continually during CPB.

**Standard 7.10:** Venous oxygen saturation shall be monitored continually during CPB.

**Guideline 7.1:** Carbon dioxide removal should be monitored continually during CPB.

**Guideline 7.2:** Arterial oxygen saturation should be monitored continually during CPB.

**Guideline 7.3:** The following patient pressures should be monitored during CPB:

- Central venous pressure; and/or
- Pulmonary artery blood pressure.

**Guideline 7.4:** Continuous in-line blood gas monitoring should be used during CPB.

**Guideline 7.5:** Cerebral oximetry should be used during CPB.

**Guideline 7.6:** Arterial blood flow should be monitored continually at a point in the CPB circuit where it accurately reflects the flow delivered to the patient during CPB (*e.g.*, distal to intracircuit shunts).

**Standard 8: Anticoagulation**

**Standard 8.1:** The perfusionist, in collaboration with the physician-in-charge, shall define the intended treatment algorithm for anticoagulation management (heparin) and an alternative algorithm for when heparin is not suitable, including acceptable ranges for activated clotting time (ACT).

**Standard 8.2:** The perfusionist shall work closely with the surgical care team to monitor and treat the patient's anti- coagulation status before, during, and after the CPB period.

**Guideline 8.1:** The surgical care team should determine the target ACT by considering relevant factors, including variability in the measurement of ACT attributed to the device's performance characteristics.

**Guideline 8.2:** Patient-specific initial heparin dose should be determined by one of the following methods:

- Weight;
- Dose-response curve (automated or manual);
- Blood volume; or
- Body surface area.

**Guideline 8.3:** Anticoagulation monitoring should include the testing of ACT. Additional monitoring tests may include:

- Heparin level measurement, *e.g.*, heparin/protamine titration or unfractionated heparin level;
- Partial thromboplastin time;
- Thromboelastograph;
- Thrombin time; and/or
- Anti-Xa.

Any point-of-care (POC) device should be used under the hospital clinical laboratory policies. The Clinical Laboratory Director is responsible for all POCs tests performed within the institution. The clinical laboratory should provide documented standard procedures to all sites using POC devices for guidance on its pre-analytical, analytical and post-analytical phases, including:

- systematic recording and release of interim results;
- procedure for potentially critical lab test results;
- systematic review of results and release of reports by qualified professional.

The clinical laboratory should keep records for the quality control program as well as the standards procedures to perform them. The clinical laboratory should promote and maintain records of its ongoing users' education process for POC equipments^[12]^.

**Guideline 8.4:** Additional doses of heparin during CPB should be determined by using an ACT and/or heparin/ protamine titration. Note: in patients requiring longer CPB times (>2 to 3 hours), maintenance of higher and/or patient-specific heparin concentrations during CPB may be considered to reduce hemostatic system activation, reduce consumption of platelets and coagulation proteins, and to reduce blood transfusion (Class IIb, Level of evidence B)^[13]^.

**Guideline 8.5:** Heparin reversal should be confirmed by ACT and/or heparin/protamine titration.

**Standard 9: Blood Management**

**Standard 9.1:** The perfusionist shall participate in efforts to minimize hemodilution and avoid unnecessary blood transfusions^[13]^.

**Standard 9.2:** The perfusionist shall minimize the CPB circuit size to reduce prime volume^[13]^.

**Standard 9.3:** The perfusionist shall calculate and communicate to the surgical team before initiating CPB a patient's predicted postdilutional hemoglobin or hematocrit.

**Guideline 9.1:** Blood management efforts should include the following^[13]^:

- Participate in preoperative briefings (discussions) with the surgical care team (Standard 5.1) regarding transfusion strategies and target hematocrit values.
- Participation in a multidisciplinary blood management team.
- Minimize hemodilution by:
- Matching the size of the CPB circuit to the size of the patient;
- Autologous priming of CPB circuit, including retrograde arterial and venous antegrade priming;
- Biocompatible coating on the surface of all CPB components;
- Perioperative blood cell recovery and reinfusion after being appropriately processed; and CPB circuit blood salvage at the end of the procedure.

**Guideline 9.2:** Point-of-care hemostasis monitoring should be used to minimize blood loss.

Monitoring may include:

- International normalized ratio;
- Partial thromboplastin time;
- Prothrombin time;
- Thrombin time;
- Thromboelastography/thromboelastometry;
- Platelet count; and/or
- Platelet function analysis.

**Standard 10: Gas Exchange**

**Standard 10.1:** Gas exchange shall be maintained during CPB according to protocol accounting for:

- The individual patient characteristics/risk profile;
- Oxygenator type, design, and instructions for use; and
- Blood flow, temperature, and metabolic demand.

**Standard 10.2:** Devices used to measure gas exchange shall be calibrated according to the manufacturer's instructions for use.

**Standard 10.3:** Blood gas analysis shall be performed and recorded according to protocol.

**Guideline 10.1:** Point-of-care testing should be considered to provide accurate and timely information for blood gas analysis^[14]^.

**Guideline 10.2:** Oxygen delivery and consumption calculations should be used to evaluate and optimize gas exchange^[15]^:

Oxygen delivery: DO_2_ = 10 CI CaO_2_

Oxygen consumption: VO_2_ = 10 CI (CaO_2_ - CvO_2_)

Where:

CaO_2_ (arterial oxygen content) = (Hb x 1.36 x SaO_2_) + (0.0031 x PaO_2_)

and

CvO2 (mixed venous oxygen content) = (Hb x 1.36 x SvO_2_) + (0.0031 x PvO_2_)

CI = cardiac index

HB = hemoglobin

PaO_2_ = partial pressure of oxygen in arterial blood

PvO_2_ = partial pressure of oxygen in venous blood

SaO_2_ = arterial oxygen saturation

SvO_2_ = venous oxygen saturation

**Guideline 11.1:** Variance from intended and targeted blood flow should be communicated to the physician-in-charge.

**Guideline 11.2:** Appropriate blood flow rate should be determined by evaluation of:

- Acid base balance
- Base excess;
- Anesthetic level;
- Arterial blood pressure;
- Cerebral oximetry;
- Lactate burden; and
- Oxygen delivery and consumption (refer to Guideline 10.2 for formulae).
- Venous pO_2_
- Arterial pO_2_
- Hemoglobin concentration
- Arterial oxygen saturation
- Systemic vascular resistance (SVR);
- Temperature; and
- Venous oxygen saturation.

**Standard 12: Blood Pressure**

**Standard 12.1:** The perfusionist, in collaboration with the physician-in-charge, shall define and communicate the intended treatment algorithm for blood pressure management before CPB, including acceptable ranges for blood pressure. Obs: in many circumstances, the physician-in-charge may direct the perfusionist to modify the intended blood pressure management to address circumstances occurring during the CPB procedure.

**Standard 12.2:** The perfusionist shall work closely with the surgical care team to maintain blood pressure according to protocol during CPB.

**Guideline 12.1:** Variance from intended and targeted blood pressure should be documented and communicated to the physician-in-charge to allow for changes in the blood pressure management plan.

**Standard 13: Quality Assurance and Improvement**

**Standard 13.1:** The perfusionist shall actively participate in both institutional and departmental quality assurance and improvement programs.

**Guideline 13.1:** The perfusionist should collect data regarding conduction of the perfusion through a clinical registry or database.

**Guideline 13.2:** The perfusionist should use this data for quality control and improvement projects^[16,17]^.

**Guideline13.3:** The perfusionist should evaluate the postoperative period of the patient in a standard form (Appendix E), storing data for periodic evaluation of perfusion in his service^[18]^.

**Guideline 13.4:** Specific and periodic meetings should be held in his service for the review of avoidable errors occurring in his daily practice.

**Guideline 13.5:** Any adverse events shall be notified in writing to the responsible sector, which shall forward them to the regulatory agencies and other competent areas after their verification. The service should encourage notifications to be always carried out, establishing a direct line of communication between the team and the risk management department, guaranteeing their confidentiality^[19]^.

**Standard 14: Maintenance**

**Standard 14.1:** The perfusionist shall assure that properly maintained and functioning equipment is used in the conduct of CPB, including (but not limited to):

- Heart-lung machine Pumps
- Timers
- Pressure monitors
- Temperature monitors
- Low-level alarm
- Air bubble detector(s)
- Blood flow sensors
- Heater/cooler
- Anesthetic vaporizer
- Oxygen blender/flow meter
- Oxygen analyzer
- Ancillary equipment
- Intra-arterial blood pressure
- Vascular assist device
- Cell salvage device

**Standard 14.2:** Preventive maintenance on perfusion equipment shall be performed and documented on a regularly scheduled basis by the perfusion team and/or appropriately trained and qualified biomedical engineering staff. Any or all of the following may determine the interval of such maintenance:

- Manufacturer recommendations;
- External accrediting agency guidelines; and/or
- Institutional requirements.

**Note:** In the case of consigned equipment, the owner of the CPB machine is responsible for maintaining the perfusion set, and all liabilities and legal issues will be assigned to it. In the case of an adverse event resulting from the use of this equipment, even if the equipment is proven to be defective, even with adequate maintenance and not related to improper use by the perfusionist, the owner of the equipment and not the institution shall be held liable.

Therefore, there should be an updated document for each of the equipment used, with dates and details of preventive and corrective maintenance and that should be filed in the perfusion service/department or clinical engineering unit of the institution^[20]^.

**Standard 14.3:** The organization shall have a written procedure for perfusion equipment failures^[21]^.

**Standard 14.4:** Appropriate back-up perfusion supplies shall be readily available. Obs: when CPB machine is not property of the institution, the equipement's owner will be responsible for the replacement, and has legal responsibility for this action.

**Standard 15: Duty Hours**

**Standard 15.1:** The perfusionist can be hired by the hospital or through a medical services company, respecting the labor laws according to the signed agreement.

**Standard 15.2:** It is briefly forbidden for the perfusionist to be engaged to perform perfusion and perform another function in the service with the same labor contract.

**Guideline 15.1** The perfusionist should receive a minimum of 8 hours of rest period for each consecutive 16-hour work period^[22-24]^.

**REFERENCES**

1. SBCEC: Sociedade Brasileira de Circulação Extracorpórea. Normas Brasileiras para o Exercício da Especialidade de Perfusionista em Circulação Extracorpórea [Internet]. Campinas (SP): SBCEC; 2017 [cited 2018 Feb 08]. 54 p. Available from: http://www.sbcec.com.br/br/images/pdf/normas_brasileiras_cec.pdf

2. American Society of ExtraCorporeal Technology [Internet]. American Society of ExtraCorporeal Technology Standards and Guidelines for Perfusion Practice (5/23/2017) [Internet]. Chicago (IL): American Society of ExtraCorporeal Technology; c2019 [cited 2019 Feb 08]. Available from: http://www.amsect.org/p/cm/ld/fid=1617

3. Ministério do Trabalho e Emprego (MT), Gabinete do Ministro. NR 32 - Segurança e saúde no trabalho em serviços de saúde [Internet]. Brasília: República Federativa do Brasil; 2005 [cited 2019 Feb 24]. Nov 11. Available from: http://www.fiocruz.br/biosseguranca/Bis/manuais/legislacao/NR-32.pdf

4. Haynes AB, Weiser TG, Berry WR, Lipsitz SR, Breizat AH, Dellinger EP, et al. A surgical safety checklist to reduce morbidity and mortality in a global population. N Engl J Med. 2009 Jan 29;360(5):491-9. doi: 10.1056/NEJMsa0810119.

5. McCarthy D, Chase D, Issues Research. Advancing patient safety in the U.S. Department of veterans affairs. Commonweath Fund [Internet]. 2011 [cited 2019 Feb 24]; 1477(9):1-32. Available from: https://www.commonwealthfund.org/sites/default/files/documents/___media_files_publications_case_study_2011_mar_1477_mccarthy_va_case_study_final_march_v2.pdf

6. Workd Alliance for Patient Safety. WHO surgical safety checklist and implementation manual [Internet]. Genova: WHO; 2008. Available from: [cited 2019 Feb 24]. HYPERLINK "http://www.who.int/patientsafety/safesurgery/ss_checklist/en/"www.who.int/patientsafety/safesurgery/ss_checklist/en/

7. American College of Surgeons. Statement on distractions in the operating room [Internet]. Chicago (IL): American College of Surgeons; Out 2016. Available from: https://www.facs.org/about-acs/statements/89-distractions

8. Cunha C, Moraes F, Monteiro V, Feitosa F, Silva I. Avaliação microbiológica dos aparelhos celulares de profissionais do Bloco Cirúrgico em um Hospital beneficente. Rev Epidemiol Control Infecc. 2016;6(3): 120-4. doi:HYPERLINK "https://doi.org/10.17058/reci.v6i3.6717" 10.17058/reci.v6i3.6717

9. Conselho Regional de Enfermagem de Santa Catarina (COREN). Parecer Coren/SC n. 005/CT/2015. Assunto: Uso de aparelho celular no ambiente hospitalar [Internet]. Florianopolis (SC): Coren; 2016 [cited 2019 Feb 24]. 4 p. Available from: http://www.corensc.gov.br/wp-content/uploads/2016/08/Parecer-Técnico-005-2016-Uso-de-aparelho-celular-no-ambiente-hospitalar.pdf

10. Wadhera RK, Parker SH, Burkhart HM, Greason KL, Neal JR, Levenick KM, et al. Is the "sterile cockpit" concept applicable to cardiovascular surgery critical intervals or critical events? The impact of protocol-driven communication during cardiopulmonary bypass. J Thorac Cardiovasc Surg. 2010 Feb;139(2):312-9. doi: 10.1016/j.jtcvs.2009.10.048.

11. Whyte S, Cartmill C, Gardezi F, Reznick R, Orser BA, Doran D, Lingard L. Uptake of a team briefing in the operating theatre: a Burkean dramatistic analysis. Soc Sci Med. 2009 Dec;69(12):1757-66. Doi: 10.1016/j.socscimed.2009.09.054

12. de Vries EN, Prins HA, Crolla RM, den Outer AJ, van Andel G, van Helden SH, et al. Effect of a comprehensive surgical safety system on patient outcomes. N Engl J Med. 2010 Nov 11;363(20):1928-37. doi: 10.1056/NEJMsa0911535.

13. Ministério da Saúde (MS), Agência Nacional de Vigilância Sanitária (ANVISA). Resoluçao RDC n. 302, de 13 de outubro de 2005. Dispõe sobre Regulamento Técnico para funcionamento de Laboratórios Clínicos [Internet]. Brasília: Republica Federativa do Brasil; 2005 [cited 2019 Feb 24]. Out 14; 2005. Available from: http://portal.anvisa.gov.br/documents/10181/2718376/RDC_302_2005_COMP.pdf/7038e853-afae-4729-948b-ef6eb3931b19

14. Society of Thoracic Surgeons Blood Conservation Guideline Task Force, Ferraris VA, Brown JR, Despotis GJ, Hammon JW, Reece TB, Saha SP, Song HK, Clough ER; Society of Cardiovascular Anesthesiologists Special Task Force on Blood Transfusion, Shore-Lesserson LJ, Goodnough LT, Mazer CD, Shander A, Stafford-Smith M, Waters J; International Consortium for Evidence Based Perfusion, Baker RA, Dickinson TA, FitzGerald DJ, Likosky DS, Shann KG. 2011 update to the Society of Thoracic Surgeons and the Society of Cardiovascular Anesthesiologists blood conservation clinical practice guidelines. Ann Thorac Surg. 2011 Mar;91(3):944-82. doi: 10.1016/j.athoracsur.2010.11.078.

15. Nichols JH, editor. Evidence-based practice for point-of-care testing [Internet]. Whashigton (DC): AACC; 2006 [cited 2019 Feb 24]. 203 p. Available from: https://www.aacc.org/SiteCollectionDocuments/NACB/LMPG/POCT/POCT%20Entire%20LMPG.pdf

16. de Somer F, Mulholland JW, Bryan MR, Aloisio T, Van Nooten GJ, Ranucci M. O2 delivery and CO2 production during cardiopulmonary bypass as determinants of acute kidney injury: time for a goal-directed perfusion management? Crit Care. 2011 Aug 10;15(4):R192. doi: 10.1186/cc10349.

17. Warren CS, DeFoe GR, Groom RC, Pieroni JW, Groski CS, Morse CB, et al. Variation in arterial inflow temperature: a regional quality improvement project. J Extra Corpor Technol. 2011 Jun;43(2):58-63.

18. Baker RA, Newland RF, Fenton C, McDonald M, Willcox TW, Merry AF; Perfusion Downunder Collaboration. Developing a benchmarking process in perfusion: a report of the Perfusion Downunder Collaboration. J Extra Corpor Technol. 2012 Mar;44(1):26-33.

19. Asociación Española de Perfusionistas (AEP). Manual de Calidad em Perfusión [Internet]. Madrid: Asociación Española de Perfusionistas (AEP); [date unknwon] [cited 2019 Feb 24]. 27 p. Available from: https://www.aep.es/comisiondocumentos/7/Manual_de_calidad.pdf

20. Agência Nacional de Vigilância Sanitária (ANVISA). Nota Técnica GVIMS/GGTES/ANVISA N.1/2005. Orientações gerais para a notificação de eventos adversos relacionados à assistência à assistência à saúde. Revisada 28 de Agosto de 2018. [Internet]. Brasília: ANVISA; 2018 [cited 2019 Feb 24]. 37 p. Available from: https://www20.anvisa.gov.br/segurancadopaciente/index.php/alertas?task=callelement&format=raw&item_id=419&element=a94a4264-a31c-42d6-91d7-464889cc6e50&method=download

21. Ministerio da Saúde (MS), Agência Nacional de Vigilância Sanitária (ANVISA). Resolução de Direotria Colegiada - RDC n. 2, de 24 de janeiro de 2010. Dispõe sobre o gerenciamento de tecnologias em saúde em estabelecimentos em saúde [Internet]. Brasilia: Republica Federativa do Brasil; 2010 [cited 2019 Feb 24]. Jan 26. Available from: http://portal.anvisa.gov.br/documents/10181/2718376/RDC_02_2010_COMP.pdf/0a8661c8-9323-4747-b103-6e83c4ff41cd

22. Lipschultz A. Environment of Care Essentials for Health Care. Biomed Instruct Technol. 2012;46(5):384.

23. The Society of Clinical Perfusion Scientists of Great Britain and Ireland, The College of Clinical Perfusion Scientists of Great Britain and Ireland. Standards of Practice Document. Standards of practice document [Internet]. London: The Society of Clinical Perfusion Scientists of Great Britain & Ireland; c 2018. Available from: HYPERLINK "http://www.scps.org.uk/index.php?option=com_content&task=view&id=25&Itemid=40"www.scps.org.uk/index.php?option=com_content&task=view&id=25&Itemid=40.

24. Accreditation Council for Graduate Medical Education (ACGME). Policies and procedures [Internet]. Chicago (IL): ACGME; Sep 29, 2018 [cited 2019 Feb 24]. Available at: https://www.acgme.org/Portals/0/PDFs/ab_ACGMEPoliciesProcedures.pdf

*Note: "The SBCCV/SBCEC Comprehensive Standards and Guidelines for Perfusion Practice in Brazil have been modified and adapted, following Brazilian regulatory agencies' policies and recommendation, from The American Society of Extracorporeal Technology (AmSECT) Standards and Guidelines[2] and translated to Portuguese"

## Appendix A

**Patient Information**

1. Medical record number
2. Patient surname, first name
3. Demographics
  - Age (date of birth)
  - Gender
  - Height
  - Weight
  - Body surface area (BSA)
4. Blood type
5. Laboratory data
  - Hemoglobin/hematocrit
  - Predicted hematocrit on bypass
  - White blood cell count
  - Platelet count
  - Activated partial prothrombin time
  - Sodium
  - K+
  - Blood urea nitrogen/creatine
  - Glucose
  - Other relevant laboratory values
6. Patient allergies
7. Planned procedure
8. Medical history/risk factors (recommended)
  - Cardiovascular
  - Pulmonary
  - Renal
  - Neurologic
  - Gastrointestinal/endocrine

## Appendix B

**Information Sufficient to Accurately Describe the Procedure, Personnel, and Equipment**

1. Date of procedure
2. Type of procedure
3. Perfusionist(s) names
4. Surgeon(s) name
5. Anesthesiologist(s) name
6. Nurse(s) name
7. Operating room number
8. Comments/events (recommended)
9. Equipment
  - Heart-lung machine
  - Cell salvage (autotransfusion) device
  - Heater/cooler
  Note: Items A-C are often uniquely identified (*e.g*., Pump 1, 2, 3, etc.) The related serial numbers for each component (*e.g*., roller pumps, vaporizer, blender, etc.) are documented and stored locally.
10. Disposables
  - Oxygenator
  - Cardiotomy reservoir
  - Tubing pack/arterial line filter
  - Centrifugal pump head
  - Cardioplegia delivery system
  - Cell salvage (autotransfusion)
  - Ultrafiltration device
  - Arterial cannula
  - Venous cannula
  - Cardioplegia cannulae
  - Sump/vent(s)
  Note: Manufacturer, model, serial, and/or lot numbers should be documented with items a-k.

## Appendix C

**Patient Physiological and Perfusionist Practice Parameters Documented at a Frequency Determined by Institutional Protocol**

1. Blood flow rates (RPM)
2. Arterial blood pressure
3. Arterial line pressure
4. Central venous/pulmonary artery pressure
5. Vacuum assist venous return (VAVR)
  - VAVR pressure
  - Venous inlet pressure (VIP)
6. Arterial/venous blood gases
7. Venous oxygen saturation
8. Patient temperatures, including:
  - Patient core (at least one)
    - Nasopharyngeal
    - Bladder
    - Esophageal
    - Rectal
    - Tympanic
    - Myocardium (optional)
9. Cardiopulmonary bypass temperatures:
  - Venous return blood
  - Arterial blood inflow
  - Water bath(s) (optional)
10. Oxygenator gases including gas flow rate and concentration(s)
11. Input fluid volumes including:
  - Prime
  - Blood products
  - Asanguineous fluids
  - Cardioplegic solution
  - Autologous components
12. Cardioplegia
  - Solution (ratio)
  - Route
  - Flow
  - Pressure
  - Temperature
  - Volume
13. Output fluid volumes, including:
  - Urine output
  - Ultrafiltrate
14. Medications and/or inhalational anesthetic agents administered through extracorporeal circuit

## Appendix D

**Blood Gas, Electrolyte, and Anticoagulation Monitoring Results**

1. Blood gases
  - pO_2_
  - pCO_2_
  - pH
  - Base excess
  - Bicarbonate concentration
  - Saturation
  - Potassium concentration
  - Ionized calcium concentration i. Sodium concentration
  - Lactate
  - Glucose
  - Hemoglobin/hematocrit
2. Activated clotting times (ACTs) and/or heparin/ protamine assay results and/or thromboelastography results.

## Appendix E

**Postoperative Evaluation of Patients Submitted to Extracorporeal Circulation**

1. Name
2. Patient's hospital identification number
3. Date of surgery
4. Date/Time of ICU admission
5. Values of CK, CKMB and Troponin at 6hs, 12hs and 24hs
6. Ejection fraction in the postoperative period.
7. Vasoactive drugs dose used at 6hs, 12hs and 24hs
8. Water balance every 6 hours until completing 48 hours of ICU
9. Central venous pressure every 6 hours, until completing 48 hours of ICU
10. Use of blood products
11. Extubation Time
12. Date of ICU discharge

## Appendix F

**Checklist**

The checklist should check, at least:

- Integrity and operation of the CEC machine and heat exchanger.
- Operation of gas systems and connections to the oxygenator.
- Correct identification of the patient.
- Availability of blood products.
- Composition of the perfusate.
- Composition and preparation of cardioplegia.
- Cannulas that will be used.
- All circuit connections.
- Calibration and direction of the rollers.
- Calibration of pressure, flow and bubble sensors and alarms.
- Calibration of cardioplegia pressure sensors.
- Calibration and connection of the gas monitor.
- Availability of disposable materials and emergency equipment (hand crank, emergency light, etc.)
- Dose and administration of heparin.
- Outcome of ACT before starting ECC.
- Availability of drugs, needles, syringes, and serum and blood infusion kits for use during CPB.

## Appendix 2

**SBCCV/SBCEC Padrões e Diretrizes Abrangentes para a Prática de Perfusão no Brasil***

**Standard 1: Desenvolvimento de um protocolo institucional próprio**

**Standard 1.1:** A instituição ou o provedor de serviços de perfusão deve desenvolver e implementar os procedimentos operacionais padrão (protocolo, POP) para cada um dos procedimentos realizados como um mecanismo de aplicação das recomendações desse documento para a prática clínica.

**Standard 1.2:** O protocolo deverá ser:

- Aprovado pelo(a) chefe da cirurgia cardíaca ou seu/sua designado(a), diretor(a) da perfusão ou equivalente ou outra pessoa ou comitê de relevância hierárquica de decisão das práticas clínicas institucionais.
- Revisto e reavaliado, ao menos anualmente ou com maior frequência quando necessário.

**Guideline 1.1:** Mudanças no protocolo podem ser feitas a critério da Equipe Cirúrgica e devem ser documentadas na ficha de perfusão.

**Standard 2: Qualificação, Competência e Pessoal de Apoio**

**Standard 2.1:** A circulação extracorpórea só deve ser ralizada por profissional formado nas profissões reconhecidas pela Sociedade Brasileira de Circulação Extracorpórea (SBCEC) e pela Sociedade Brasileira de Cirurgia Cardiovasccular (SBCCV) como competentes para realizar o procedimento; ter pós-graduação reconhecida pelo MEC com carga horária mínima descrita no artigo 12 das Normas Brasileiras para o Exercício da Especialidade de Perfusionista em Circulação Extracorpórea ou Título de Especialista da SBCEC^[1]^, validado por esta Sociedade; ou profissionais que se enquadram no parágrafo único do artigo 2º da Norma acima citada.

**Standard 2.2:** As competências do perfusionista devem ser avaliadas anualmente com o intuito de averiguar se estão em conformidade com os protocolos departamentais.

**Standard 2.3:** O perfusionista deve assistir, participar e se envolver em cursos de educação continuada relacionados à perfusão ao menos uma vez ao ano.

**Standard 2.4:** O pessoal de apoio deve estar disponível no local para auxiliar o perfusionista durante os procedimentos necessários na condução da CEC.

**Guideline 2.1:** Um indivíduo que se formar em um programa credenciado de educação em perfusão deve preencher todos os requisitos para a certificação da SBCEC.

**Guideline 2.2:** Um processo padronizado deve ser desenvolvido e seguido para identificar, orientar e educar a equipe de suporte de forma a garantir que todos tenham conhecimento geral das funções desempenhadas pelo perfusionista, fluxo cirúrgico e localização dos itens essenciais e auxiliares necessários durante a CEC. A equipe de apoio inclui equipes de perfusionistas, enfermagem, técnicos e pessoal administrativo.

**Guideline 2.3:** Um programa estruturado para educar, treinar e avaliar a equipe de perfusão, ao menos anualmente, deve ser desenvolvido e seguido.

**Guideline 2.4:** É recomendada a existência de dois perfusionistas por procedimento, garantindo maior segurança ao procedimento.

**Guideline 2.5:** Utilização de equipamentos de proteção individual. Durante a condução da CEC o perfusionista deve utilizar equipamentos de proteção individual (EPI), como máscaras, óculos e luvas de procedimento. As luvas devem ser trocadas após a coleta de amostra, após a troca de solução (bolsa de sangue) do equipo ou sempre que estiver com respingo de sangue^[2]^.

**Standard 3: Ficha (Registro) de Perfusão**

**Standard 3.1:** A ficha (registro) de perfusão (escrita e/ou eletrônica) para cada procedimento de CEC deve ser incluída como parte do prontuário médico do paciente. O registro de perfusão deve ser mantido e armazenado de acordo com a política da instituição para a retenção de registros médicos (prontuário) do paciente.

**Standard 3.2:** A ficha (registro) deve incluir:

- Informações do paciente, incluindo dados demográficos e fatores de risco pré-operatório (Appendix A).
- Informações necessárias para descrever com precisão o procedimento, pessoal e equipamento utilizado (Appendix B).
- Parâmetros fisiológicos do paciente documentados de acordo com a frequência determinada pelo protocolo institucional (Appendix C).
- Gases sanguíneos e resultados da monitoração da anticoagulação (Appendix D).
- o Assinatura e carimbo do perfusionista (e de todos os demais perfusionistas de apoio) que participaram do procedimento.

**Guideline 3.1:** Deverá haver uma ficha (registro) de perfusão (escrita e/ou eletrônica) para cada procedimento de CEC. A ficha de perfusão deve incluir um espaço de texto livre para registro de comentários, incluindo ordens verbais dadas pela equipe médica pertinentes à perfusão realizada.

**Guideline 3.2:** O registro de perfusão deve incluir as assinaturas do(s) médico(s) responsável(is) pela supervisão da CEC.

**Guideline 3.3:** Os dados brutos (por exemplo, fluxo sanguíneo, pressão e valores de temperatura) contidos em bancos de dados de perfusão eletrônica devem ser armazenados por um período de tempo definido pela política institucional referente à retenção de registros médicos eletrônicos de pacientes.

**Standard 4: *Checklist***

**Standard 4.1:** O perfusionista deve utilizar um *checklist* para cada perfusão realizada^[4]^.

**Standard 4.2:** O *checklist* deve ser preenchido, incluído e anexado ao prontuário do paciente.

**Guideline 4.1:** O perfusionista deve fazer o *checklist* lendo-o em voz alta a outro perfusionista e marcando cada um dos itens após a confirmação de que aquela ação, definida item a item, foi realizada^[4]^. O preenchimento do *cheklist* deve ser realizado por duas pessoas, sendo uma delas o perfusionista principal responsável pela condução da CEC durante o período intraoperatório. Nos serviços em que não há a disponibilidade de outro profissional perfusionista, deve ser adotada uma rotina sistemática de verificação dos itens contidos no *checklist*, com o objetivo de minimizar a ocorrência de eventos adversos.

**Guideline 4.2:** O perfusionista deve utilizar o *checklist* ao longo de todo o período perioperatório (p. ex., montagem da máquina de CEC, pré-CEC, fase inicial da perfusão, antes da saída da perfusão, após a perfusão e/ou qualquer retorno à CEC).

**Guideline 4.3:** O perfusionista deve utilizar o *checklist* para os demais serviços auxiliares à perfusão (isto é, recuperação sanguínea através de "*cell salvage*", balão intra-aórtico, oxigenação por membrana extracorpórea - *extracorporeal membrane oxygenation* - ECMO, entre outros).

**Standard 5: Comunicação**

**Standard 5.1:** Um plano de perfusão individualizado e especifíco ao paciente deve ser preparado e comunicado à equipe cirúrgica tanto no "*briefing*" quanto antes do início do procedimento^[5]^.

**Guideline 5.1:** O uso de aparelhos de telefone celular em sala cirúrgica deve ser evitado e em CEC deve ser proibido, visto que é um fator de distração e predisponente para riscos ao paciente. Além disso, a grande maioria está contaminada por bactérias potencialmente infectantes, comprometendo a qualidade e segurança da assistência. Quando seu uso for inevitável, os aparelhos devem ser higienizados previamente, conforme protocolo de antissepsia da instituição^[6-8]^.

**Guideline 5.2:** A comunicação padronizada por meio de protocolos específicos (p. ex., *closed loop communication*) deve ser utilizada para o reconhecimento dos comandos verbais, verificação de conteúdo e redução de ambiguidade^[9-11]^.

**Guideline 5.3:** O perfusionista principal deverá participar do "*debrief*" pós-operatório com toda a equipe cirúrgica.

**Standard 6: Dispositivos de Segurança**

**Standard 6.1:** O monitoramento de pressão da linha arterial, do sistema de administração de cardioplegia e do reservatório venoso (quando a drenagem venosa assistida por vácuo for utilizada) deve ser realizado e documentado durante a CEC.

- O monitor de pressão deve ser servorregulado para o controle do rolete arterial e de cardioplegia de acordo com a pressão da linha arterial e da linha de cardioplegia, permitindo a interrupção do fluxo de ambas.
- O monitor de pressão deve incluir um alarme audiovisual.

**Standard 6.2:** Detector de bolhas deve ser utilizado durante a CEC.

- O detector de macrobolhas deve ser usado para controlar a bomba arterial ou para permitir a interrupção do fluxo sanguíneo arterial.
- O sistema detector deve incluir um alarme audível e visual e ser posicionado de acordo com as instruções do fabricante para uso do oxigenador, ligado e testado antes de cada procedimento, de forma a permitir a identificação e ação a tempo de evitar sua passagem para o paciente.

**Standard 6.3:** Um sensor de nível deve ser utilizado durante a CEC quando utilizado um reservatório rígido (*hard-shell venous reservoir*) no circuito.

- O sensor de nível deve ser servorregulado para controlar a bomba arterial ou para permitir a interrupção do fluxo sanguíneo arterial.
- O sensor de nível deve incluir um alarme audível e visual e ser posicionado de acordo com as instruções do fabricante para permitir um tempo de reação apropriado e um volume operacional seguro.

**Standard 6.4:** O monitoramento da temperatura da saída arterial do oxigenador deve ser utilizado durante os procedimentos de CEC.

- O sensor de temperatura deve incluir um alarme audível e visual para a prevenção de temperaturas elevada na saída arterial do oxigenador.

**Standard 6.5:** Um filtro arterial isolado ou acoplado ao oxigenador deve ser utilizado em todo o circuito de CEC. Quando usado de forma isolada do oxigenador, deve ser utilizado no circuito depois do oxigenador e deve contar com uma linha de recirculação conectada ao reservatório de cardiotomia ou venoso. Deve dispor de um "*bypass*" que permita ao perfusionista anular o filtro em caso de obstrução ou rupture.

**Standard 6.6:** Uma válvula "*one-way*" deve ser utilizada na linha de aspiração da aorta/átrio esquerdo durante a perfusão.

**Standard 6.7:** Deve ser utilizado pelo menos um método para evitar o fluxo retrógrado para a circulação sistêmica em circuitos com bombas centrífugas. Exemplos de sistemas de evasão retrógrada podem incluir:

- Válvulas de fluxo unidirecional (*one-way flow valves*);
- Controles de redução de velocidade da bomba com mecanismos de prevenção de acionamento acidental (*hard-stop detent controls*);
- Pinças de acionamento eletrônico na linha arterial; ou
- Alarme audiovisual em caso de baixa velocidade da bomba.

**Standard 6.8:** Uma linha de eliminação de gases deve ser utilizada sempre que anestésicos inalatórios forem utilizados diretamente no circuito durante a perfusão.

**Standard 6.9:** Manivelas manuais (*hand cranks*) devem estar prontamente disponíveis durante a perfusão.

**Standard 6.10:** *Backups* (alternativas de segurança) de fonte de gás (p. ex., cilindro de oxigênio/ar comprimido) devem estar prontamente disponíveis durante a CEC.

**Standard 6.11:** A máquina de CEC deve possuir bateria incorporada ou outra fonte de energia suplementar disponível durante toda a perfusão.

**Guideline 6.1:** Um analisador de oxigênio da mistura de gás (*sweep flow*) antes da entrada de gás da membrana deve ser utilizado durante a perfusão.

**Guideline 6.2:** Um sensor de nível deve ser usado durante os procedimentos de CPB com um reservatório fechado (*soft-shell reservoir*).

- O sensor de nível deve ser servorregulado para controlar a bomba arterial ou para permitir a interrupção do fluxo sanguíneo arterial.
- O sensor de nível deve incluir um alarme sonoro e visual e ser posicionado de acordo com as instruções do fabricante para permitir um tempo de reação apropriado e um volume operacional seguro.
- O uso de um detector de bolhas de ar distal à saída do reservatório pode ser utilizado em substituição ao detector de nível.

**Guideline 6.3:** A bandeja de tubos disponibilizados para montagem do circuito deve ser disponibilizada "pré-montada", pré-conectada e em bandeja estéril separando o circuito que ficará no campo cirúrgico daquele que ficará na máquina de CEC, ofertando mais segurança ao procedimento e gerando mais rapidez na montagem do circuito.

**Standard 7: Monitoração (obs.: para ser realizado em conjunto com Standard 3)**

**Standard 7.1:** A pressão arterial do paciente deve ser monitorada de forma contínua durante toda a CEC.

**Standard 7.2:** A pressão da linha arterial do circuito de CEC deve ser monitorada durante toda a perfusão.

**Standard 7.3:** O fluxo arterial deve ser monitorado continuamente durante toda a perfusão.

**Standard 7.4:** A dose de cardioplegia, o método de infusão, a pressão de linha (cardioplegia anterógrada), a pressão do seio coronário (cardioplegia retrógrada) e os intervalos de isquemia devem ser continuamente monitorados durante a CEC.

**Standard 7.5:** A temperatura do paciente e dos dispositivos deve ser continuamente monitorada durante a CEC.

- Paciente: nasofaríngea, retal, vesical, esofágica.
- Máquina de CEC: arterial, venosa e cardioplegia.
- Trocador de calor (*heater-cooler*): temperatura da água.

**Standard 7.6:** A análise de gases sanguíneos (gasometria) deve ser monitorada continuamente ou em intervalos regulares durante a CEC (Appendix D).

**Standard 7.7:** O hematócrito (ou hemoglobina) deve ser monitorado continuamente durante a CEC.

**Standard 7.8:** A fração de oxigênio (FiO_2_) e o fluxo de gás (*sweep flow*) devem ser monitorados continuamente durante a CEC (Appendix D).

**Standard 7.9:** A porcentagem de oclusão da linha venosa do oclusor venoso (*clamp* venoso automatizado), quando disponível, deve ser monitorada continuamente durante a CEC.

**Standard 7.10:** Saturação venosa de oxigênio deve ser monitorada continuamente ou em intervalos regulares durante a CEC.

**Guideline 7.1:** A remoção de CO_2_ (etCO_2_ ou pCO_2_) deve ser continuamente monitorada durante a CEC.

**Guideline 7.2:** A saturação arterial de oxigênio (SaO_2_) deve ser continuamente monitorada durante a CEC.

**Guideline 7.3:** As seguintes pressões do paciente devem ser monitoradas durante a CEC:

- Pressão venosa central (PVC); e/ou
- Pressão da artéria pulmonar.

**Guideline 7.4:** Os gases sanguíneos devem ser continuamente monitorados (in-line) durante a CEC.

**Guideline 7.5:** A oximetria cerebral (NIRS) deve ser utilizada durante a CEC sempre que dísponível.

**Guideline 7.6:** O fluxo sanguíneo arterial deve ser monitorado continuamente em um ponto no circuito no qual reflete com precisão o fluxo entregue ao paciente durante a CEC (p. ex., distal à "*purge line*").

**Standard 8: Anticoagulação**

**Standard 8.1:** O perfusionista, em colaboração com o cirurgião responsável, deve definir o algoritmo pretendido para o manejo da anticoagulação (heparina) e um algoritmo alternativo para a heparinização não adequada, incluindo intervalos aceitáveis para o tempo de coagulação ativada (TCA).

**Standard 8.2:** O perfusionista deve trabalhar em estreita colaboração com a equipe de cuidados cirúrgicos na monitoração e no tratamento do estado de coagulação do paciente antes, durante e após a CEC.

**Guideline 8.1:** A equipe cirúrgica deve determinar o alvo do TCA considerando fatores relevantes, incluindo variabilidade em sua medida atribuída às características de desempenho do dispositivo.

**Guideline 8.2:** A dose inicial de heparina específica para o paciente deve ser determinada por um dos seguintes métodos:

- Peso;
- Curva de dose-resposta (automatizada ou manual);
- Volume sanguíneo; ou
- Superfície corpórea.

**Guideline 8.3:** O monitoramento da anticoagulação deve incluir o TCA, devendo-se realizar um TCA inicial, outro após a protamina e, durante a CEC, pelo menos um a cada 30 minutos. Testes de monitoramento adicionais podem incluir:

- Nível de heparina (p. ex., titulação de heparina/protamina ou nível de heparina não fracionado);
- Tempo de tromboplastina parcial ativada (TTPa);
- Tromboelastograma;
- Tempo de trombina (TP); e/ou
- Anti-Xa.

A execução dos testes laboratoriais remotos - TLR (*point-of-care*) deve estar vinculada a um laboratório clínico do hospital. O responsável técnico pelo laboratório clínico é responsável por todos os TLR realizados dentro da instituição. O laboratório clínico deve disponibilizar, nos locais de realização de TLR, procedimentos documentados com orientações sobre suas fases pré-analítica, analítica e pós-analítica, incluindo:

- Sistemática de registro e liberação de resultados provisórios.
- Procedimento para resultados potencialmente críticos.
- Sistemática de revisão de resultados e liberação de laudos por profissional habilitado.

O laboratório clínico deve manter registros dos controles da qualidade, bem como procedimentos para sua realização.O laboratório clínico deve promover e manter registros de seu processo de educação permanente para os usuários dos equipamentos de TLR^[12]^.

**Guideline 8.4:** As doses adicionais de heparina durante a CEC devem ser determinadas usando a titulação pelo TCA e/ou heparina/protamina. Nota: em pacientes que requerem tempos de CEC mais longos (> 2 a 3 horas), pode-se considerer a manutenção de concentrações de heparina mais altas e/ou específicas para o paciente durante a CEC, para reduzir a ativação do sistema de coagulação e o consumo de plaquetas e proteínas de coagulação, assim como evitar ou reduzir a transfusão de hemoderivados (Classe IIb, Nível de evidência B)^[13]^.

**Guideline 8.5:** A reversão da heparina deve ser confirmada por TCA, tromboelastrograma e/ou titulação da heparina/protamina.

**Standard 9: Manuseio transfusional**

**Standard 9.1:** O perfusionista deve aderir às práticas recomendadas para minimizar a hemodiluição e evitar transfusões de sangue desnecessárias^[13]^.

**Standard 9.2:** O perfusionista deve minimizar o tamanho do circuito de CEC no intuito de reduzir o volume do *prime* ^[13]^.

**Standard 9.3:** O perfusionista deve calcular e comunicar à equipe cirúrgica o hematócrito/hemoglobina pós-dilucional previsto para o paciente antes de iniciar a CEC.

**Guideline 9.1:** As definições dos protocolos de transfusão de hemoderivados (*patient blood management* - PBM) devem incluir^[13]^:

- Participação do perfusionista nas discussões (*briefings*) pré-operatórias com toda a equipe cirúrgica (Standard 5.1) em relação às estratégias de transfusão e valores "alvos" de hematócrito.
- Participação do perfusionista no grupo multidisciplinar de PBM.

Minimizar a hemodiliução por meio de:

- Seleção do tamanho do circuito de CEC compatível com o tamanho do paciente.
- Preenchimento do circuito com sangue autólogo, incluindo enchimento retrógrado do circuito com sangue (*retrograde autologous priming* - RAP).
- Utilização de tubos com revestimento biocompatível/bioativos em todos os componentes de CEC.
- Recuperação perioperatória de células sanguíneas (*cell savage*) e reinfusão após processamento adequado.
- Recuperação do sangue do circuito de CEC ao final do procedimento ("*blood cell savage*" do "*prime*" do circuito).

**Guideline 9.2:** Monitoração da hemostasia por meio de "*point-of-care*" deve ser utilizada na minimização da perda sanguínea.

Essa monitoração deve incluir:

- *International normalized ratio* (INR ou RNI);
- Tempo de tromboplastina parcial ativada (TTPa);
- Tempo de protrombina (TP);
- Tempo de trombina (TT);
- Tromboelastograma;
- Contagem de plaquetas; e/ou
- Agregação plaquetária.

**Standard 10: Troca Gasosa**

**Standard 10.1:** A troca de gás deve ser mantida durante a CEC de acordo com o protocolo, considerando:

- Características individuais do paciente e perfil de risco;
- Tipo de oxigenador, *design* e instruções de uso; e
- Fluxo sanguíneo, temperatura e demanda metabólica.

**Standard 10.2:** Os dispositivos utilizados para medir a troca gasosa devem ser calibrados de acordo com as instruções de uso do fabricante.

**Standard 10.3:** A análise sanguínea dos gases (gasometria) deve ser realizada e anotada de acordo com o protocolo.

**Guideline 10.1:** Exames realizados com dispositivos "*point-of-care*" devem ser considerados para propiciar informações precisas e instantâneas para análise de gases no sangue^[14]^.

**Guideline 10.2:** O cálculo da oferta e consumo de oxigênio deve ser utilizado para avaliar e otimizar a troca gasosa^[15]^:

Oferta de oxigênio: DO_2_ = 10 x IC x CaO_2_

Consumo de oxigênio: VO_2_ = 10 x IC x (CaO_2_ - CvO_2_)

Em que:

CaO_2_ (conteúdo de oxigênio arterial) = (Hb x 1,36 x SaO_2_) + (0,0031 x PaO_2_)

e

CvO_2_ (conteúdo de oxigênio venoso misto) = (Hb x 1,36 x SvO_2_) + (0,0031 x PvO_2_)

HB = hemoglobina

IC = índice cardíaco

PaO_2_ = pressão parcial de oxigênio no sangue arterial

PvO_2_ = pressão parcial de oxigênio no sangue venoso

SaO_2_ = saturação arterial de oxigênio

SvO_2_ = saturação venosa de oxigênio

**Standard 11: Fluxo de perfusão**

**Standard 11.1:** O fluxo de perfusão alvo deve ser determinado antes de iniciar a CEC de acordo com o protocolo institucional. Obs.: superfície corpórea x índice cardíaco = fluxo sanguíneo calculado, no qual a superfície corpórea em metros quadrados é igual à raiz quadrada da altura vezes o peso dividido por 3.600 (√altura × peso/3.600), utilizando altura em centímetros (cm) e peso em kilogramas (kg).

**Standard 11.2:** O perfusionista deve trabalhar em estreita colaboração com a equipe cirúrgica na manutenção da taxa de fluxo sanguíneo definido/calculado durante a CEC.

**Guideline 11.1:** As variações do fluxo sanguíneo definido/calculado devem ser comunicadas ao médico/cirurgião responsável.

**Guideline 11.2:** O fluxo de perfusão adequado deve ser definido pela avaliação de:

- Balanço ácido-base;
- Excesso de bases (BE);
- Nível anestésico;
- Pressão sanguinea arterial;
- Oximetria cerebral (NIRS);
- Nível de lactato;
- Entrega e consumo de oxigênio (observar a fórmula no Guideline 10.2);
- pO_2_ venosa;
- pO_2_ arterial;
- Concentração de hemoglobina;
- Saturação arterial de oxigênio;
- Resistência vascular sistêmica;
- Temperatura; e
- Saturação venosa de oxigênio (SaVO_2_).

**Standard 12: Pressão arterial**

**Standard 12.1:** O perfusionista, em conjunto com o médico/cirurgião responsável, deve definir e comunicar o algoritmo de tratamento pretendido para o gerenciamento da pressão arterial antes da CEC, incluindo seus níveis aceitáveis. Obs.: em muitas circunstâncias, o médico responsável pode direcionar o perfusionista para modificar a administração da pressão arterial pretendida para atender às circunstâncias que ocorrem durante o procedimento de CEC.

**Standard 12.2:** O perfusionista deve trabalhar em conjunto com a equipe cirúrgica para manter a pressão arterial de acordo com os protocolos da CEC.

**Guideline 12.1:** A variação entre a pressão arterial definida/calculada e a atingida deve ser documentada e comunicada ao médico responsável para permitir alterações no plano de manejo da pressão arterial.

**Standard 13: Avaliação e Melhorias na Qualidade**

**Standard 13.1:** O perfusionista deve participar ativamente dos programas institucionais e departamentais de controle e melhoria da qualidade.

**Guideline 13.1:** O perfusionista deve coletar dados relativos à condução da perfusão por meio de um registro clínico ou banco de dados.

**Guideline 13.2:** O perfusionista deve usar esses dados para projetos de controle e melhoria de qualidade^[16,17]^.

**Guideline13.3:** O perfusionista deve avaliar em ficha padrão (Appendix E) o pós-operatório do paciente, armazendo dados para avaliações periódicas da perfusão no serviço^[18]^.

**Guideline 13.4:** Reuniões específicas e periódicas devem ser realizadas para a revisao de erros evitáveis que ocorram no serviço.

**Guideline 13.5:** Todo e qualquer evento adverso deve ser notificado por escrito ao setor responsável, o qual dará encaminhamento às agências regulatórias e demais orgãos competentes após apuração. O serviço deve incentivar a realização constante das notificações, estabelecendo uma linha de comunicação direta entre a equipe e a gerência de risco, garantindo seu sigilo^[19]^.

**Standard 14: Manutenção**

**Standard 14.1:** O perfusionista deve assegurar que o equipamento utilizado na condução da CEC tenha sua manutenção corretamente realizada e em perfeito estado de funcionamento, incluindo (mas não limitado a):

- Máquina de CEC
- Bombas
- Timers
- Monitores de pressão
- Monitores de temperature
- Alarme de nível
- Detector de ar/bolhas
- Sensores de fluxo sanguíneo
- Trocador de calor (*heater-cooler*)
- Vaporizador de anestésico
- Misturador de gases e fluxômetro
- Analisador de oxigênio
- Equipamentos auxiliaries
- Pressão intra-arterial
- Dispositivos de assistência circulatória
- Dispositivos de recuperação sanguínea (*cell salvage device*)

**Standard 14.2:** A manutenção preventiva do equipamento de perfusão deve ser realizada e documentada de forma regular pela equipe de perfusão e/ou equipe de engenharia biomédica apropriadamente treinada e qualificada.

Qualquer um ou todos os seguintes itens pode determinar o intervalo dessa manutenção:

- Recomendações do fabricante;
- Recomendaçõnoes das agências de acreditação; e/ou
- Protocolos institucionais.

**Obs.:** em equipamentos consignados, o proprietário da máquina de CEC é responsável pela manutenção no equipamento de perfusão, e todas as responsabilidades e questões legais serão imputadas a ele. Em caso de evento adverso decorrente do uso deste equipamento, quando comprovada falha, mesmo com provas de manutenção adequada e não relacionada ao uso indevido por parte do perfusionista, o dono do equipamento, e não a instituição, deverá ser responsabilizado.

Portanto, deve existir um documento atualizado para cada um dos equipamentos utilizados, com as datas e os detalhes de manutenção preventiva e corretivas e que deve ser arquivado na unidade de perfusão ou de engenharia clínica da instituição^[20]^.

**Standard 14.3:** A instituição deve ter o procedimento padrão por escrito documentando as falhas potenciais e ocorridas no equipamento de perfusão, bem como as condutas a serem adotadas ou correções implementadas^[21]^.

**Standard 14.4:** Os suprimentos necessários de perfusão de "*backup*" devem estar prontamente disponíveis.

**Obs.:** quando a máquina de CEC não é propriedade da instituição (em caso de equipamento consignado), o proprietário do equipamento será responsável pela substituição e terá responsabilidade legal em caso de evento adverso decorrente do uso deste equipamento, quando comprovada falha não relacionada ao uso indevido por parte do perfusionista.

**Standard 15: Horas de Serviço**

**Standard 15.1:** O perfusionista pode ser contratado pelo hospital ou por empresa de serviços médicos, respeitando as leis trabalhistas de acordo com o vínculo firmado.

**Standard 15.2:** É sumariamente proibido que o perfusionista seja contratado para realizar perfusão e exerça outra função no serviço com o mesmo contrato trabalhista.

**Guideline 15.1:** O perfusionista deve ter um mínimo de 8 horas de período de descanso para cada período de trabalho consecutivo de 16 horas^[22-24]^.

**REFERENCES**

1. SBCEC: Sociedade Brasileira de Circulação Extracorpórea. Normas Brasileiras para o Exercício da Especialidade de Perfusionista em Circulação Extracorpórea [Internet]. Campinas (SP): SBCEC; 2017 [cited 2018 Feb 08]. 54 p. Available from: http://www.sbcec.com.br/br/images/pdf/normas_brasileiras_cec.pdf

2. American Society of ExtraCorporeal Technology [Internet]. American Society of ExtraCorporeal Technology Standards and Guidelines for Perfusion Practice (5/23/2017) [Internet]. Chicago (IL): American Society of ExtraCorporeal Technology; c2019 [cited 2019 Feb 08]. Available from: http://www.amsect.org/p/cm/ld/fid=1617

3. Ministério do Trabalho e Emprego (MT), Gabinete do Ministro. NR 32 - Segurança e saúde no trabalho em serviços de saúde [Internet]. Brasília: República Federativa do Brasil; 2005 [cited 2019 Feb 24]. Nov 11. Available from: http://www.fiocruz.br/biosseguranca/Bis/manuais/legislacao/NR-32.pdf

4. Haynes AB, Weiser TG, Berry WR, Lipsitz SR, Breizat AH, Dellinger EP, et al. A surgical safety checklist to reduce morbidity and mortality in a global population. N Engl J Med. 2009 Jan 29;360(5):491-9. doi: 10.1056/NEJMsa0810119.

5. McCarthy D, Chase D, Issues Research. Advancing patient safety in the U.S. Department of veterans affairs. Commonweath Fund [Internet]. 2011 [cited 2019 Feb 24]; 1477(9):1-32. Available from: https://www.commonwealthfund.org/sites/default/files/documents/___media_files_publications_case_study_2011_mar_1477_mccarthy_va_case_study_final_march_v2.pdf

6. Workd Alliance for Patient Safety. WHO surgical safety checklist and implementation manual [Internet]. Genova: WHO; 2008. Available from: [cited 2019 Feb 24]. HYPERLINK "http://www.who.int/patientsafety/safesurgery/ss_checklist/en/"www.who.int/patientsafety/safesurgery/ss_checklist/en/

7. American College of Surgeons. Statement on distractions in the operating room [Internet]. Chicago (IL): American College of Surgeons; Out 2016. Available from: https://www.facs.org/about-acs/statements/89-distractions

8. Cunha C, Moraes F, Monteiro V, Feitosa F, Silva I. Avaliação microbiológica dos aparelhos celulares de profissionais do Bloco Cirúrgico em um Hospital beneficente. Rev Epidemiol Control Infecc. 2016;6(3): 120-4. doi:HYPERLINK "https://doi.org/10.17058/reci.v6i3.6717" 10.17058/reci.v6i3.6717

9. Conselho Regional de Enfermagem de Santa Catarina (COREN). Parecer Coren/SC n. 005/CT/2015. Assunto: Uso de aparelho celular no ambiente hospitalar [Internet]. Florianopolis (SC): Coren; 2016 [cited 2019 Feb 24]. 4 p. Available from: http://www.corensc.gov.br/wp-content/uploads/2016/08/Parecer-Técnico-005-2016-Uso-de-aparelho-celular-no-ambiente-hospitalar.pdf

10. Wadhera RK, Parker SH, Burkhart HM, Greason KL, Neal JR, Levenick KM, et al. Is the "sterile cockpit" concept applicable to cardiovascular surgery critical intervals or critical events? The impact of protocol-driven communication during cardiopulmonary bypass. J Thorac Cardiovasc Surg. 2010 Feb;139(2):312-9. doi: 10.1016/j.jtcvs.2009.10.048.

11. Whyte S, Cartmill C, Gardezi F, Reznick R, Orser BA, Doran D, Lingard L. Uptake of a team briefing in the operating theatre: a Burkean dramatistic analysis. Soc Sci Med. 2009 Dec;69(12):1757-66. Doi: 10.1016/j.socscimed.2009.09.054

12. de Vries EN, Prins HA, Crolla RM, den Outer AJ, van Andel G, van Helden SH, et al. Effect of a comprehensive surgical safety system on patient outcomes. N Engl J Med. 2010 Nov 11;363(20):1928-37. doi: 10.1056/NEJMsa0911535.

13. Ministério da Saúde (MS), Agência Nacional de Vigilância Sanitária (ANVISA). Resoluçao RDC n. 302, de 13 de outubro de 2005. Dispõe sobre Regulamento Técnico para funcionamento de Laboratórios Clínicos [Internet]. Brasília: Republica Federativa do Brasil; 2005 [cited 2019 Feb 24]. Out 14; 2005. Available from: http://portal.anvisa.gov.br/documents/10181/2718376/RDC_302_2005_COMP.pdf/7038e853-afae-4729-948b-ef6eb3931b19

14. Society of Thoracic Surgeons Blood Conservation Guideline Task Force, Ferraris VA, Brown JR, Despotis GJ, Hammon JW, Reece TB, Saha SP, Song HK, Clough ER; Society of Cardiovascular Anesthesiologists Special Task Force on Blood Transfusion, Shore-Lesserson LJ, Goodnough LT, Mazer CD, Shander A, Stafford-Smith M, Waters J; International Consortium for Evidence Based Perfusion, Baker RA, Dickinson TA, FitzGerald DJ, Likosky DS, Shann KG. 2011 update to the Society of Thoracic Surgeons and the Society of Cardiovascular Anesthesiologists blood conservation clinical practice guidelines. Ann Thorac Surg. 2011 Mar;91(3):944-82. doi: 10.1016/j.athoracsur.2010.11.078.

15. Nichols JH, editor. Evidence-based practice for point-of-care testing [Internet]. Whashigton (DC): AACC; 2006 [cited 2019 Feb 24]. 203 p. Available from: https://www.aacc.org/SiteCollectionDocuments/NACB/LMPG/POCT/POCT%20Entire%20LMPG.pdf

16. de Somer F, Mulholland JW, Bryan MR, Aloisio T, Van Nooten GJ, Ranucci M. O2 delivery and CO2 production during cardiopulmonary bypass as determinants of acute kidney injury: time for a goal-directed perfusion management? Crit Care. 2011 Aug 10;15(4):R192. doi: 10.1186/cc10349.

17. Warren CS, DeFoe GR, Groom RC, Pieroni JW, Groski CS, Morse CB, et al. Variation in arterial inflow temperature: a regional quality improvement project. J Extra Corpor Technol. 2011 Jun;43(2):58-63.

18. Baker RA, Newland RF, Fenton C, McDonald M, Willcox TW, Merry AF; Perfusion Downunder Collaboration. Developing a benchmarking process in perfusion: a report of the Perfusion Downunder Collaboration. J Extra Corpor Technol. 2012 Mar;44(1):26-33.

19. Asociación Española de Perfusionistas (AEP). Manual de Calidad em Perfusión [Internet]. Madrid: Asociación Española de Perfusionistas (AEP); [date unknwon] [cited 2019 Feb 24]. 27 p. Available from: https://www.aep.es/comisiondocumentos/7/Manual_de_calidad.pdf

20. Agência Nacional de Vigilância Sanitária (ANVISA). Nota Técnica GVIMS/GGTES/ANVISA N.1/2005. Orientações gerais para a notificação de eventos adversos relacionados à assistência à assistência à saúde. Revisada 28 de Agosto de 2018. [Internet]. Brasília: ANVISA; 2018 [cited 2019 Feb 24]. 37 p. Available from: https://www20.anvisa.gov.br/segurancadopaciente/index.php/alertas?task=callelement&format=raw&item_id=419&element=a94a4264-a31c-42d6-91d7-464889cc6e50&method=download

21. Ministerio da Saúde (MS), Agência Nacional de Vigilância Sanitária (ANVISA). Resolução de Direotria Colegiada - RDC n. 2, de 24 de janeiro de 2010. Dispõe sobre o gerenciamento de tecnologias em saúde em estabelecimentos em saúde [Internet]. Brasilia: Republica Federativa do Brasil; 2010 [cited 2019 Feb 24]. Jan 26. Available from: http://portal.anvisa.gov.br/documents/10181/2718376/RDC_02_2010_COMP.pdf/0a8661c8-9323-4747-b103-6e83c4ff41cd

22. Lipschultz A. Environment of Care Essentials for Health Care. Biomed Instruct Technol. 2012;46(5):384.

23. The Society of Clinical Perfusion Scientists of Great Britain and Ireland, The College of Clinical Perfusion Scientists of Great Britain and Ireland. Standards of Practice Document. Standards of practice document [Internet]. London: The Society of Clinical Perfusion Scientists of Great Britain & Ireland; c 2018. Available from: HYPERLINK "http://www.scps.org.uk/index.php?option=com_content&task=view&id=25&Itemid=40"www.scps.org.uk/index.php?option=com_content&task=view&id=25&Itemid=40.

24. Accreditation Council for Graduate Medical Education (ACGME). Policies and procedures [Internet]. Chicago (IL): ACGME; Sep 29, 2018 [cited 2019 Feb 24]. Available at: https://www.acgme.org/Portals/0/PDFs/ab_ACGMEPoliciesProcedures.pdf

*Nota: "SBCCV/SBCEC Padrões e Diretrizes Abrangentes para a Prática de Perfusão no Brasil" foram baseados e adaptados da publicação "The American Society of ExtraCorporeal Technology (AmSECT) Standards and Guidelines[2] e traduzidos para o português.

## Appendix A (Portuguese version)

**Informação do paciente**

1. Número do registro médico
2. Nome e sobrenome do paciente
3. Dados demográficos:
  - Idade (data de nascimento)
  - Sexo
  - Altura
  - Peso
  - Superfície corpórea (SC)
4. Tipo sanguíneo
5. Dados laboratoriais:
  - Hemoglobina/hematócrito
  - Hematócrito predito em CEC
  - Leucócitos
  - Contagem de plaquetas
  - Tempo de protrombina parcial ativada (TTPa)
  - Sódio
  - Potássio
  - Ureia/creatinina
  - Glicemia
  - Outros valores laboratoriais relevantes
6. Alergias do paciente
7. Procedimento cirúrgico planejado
8. História médica e fatores de risco (recomendado):
  - Cardiovascular
  - Pulmonar
  - Renal
  - Neurológico
  - Gastrointestinal/endócrino

## Appendix B (Portuguese version)

**Informações básicas para descrição detalhada do procedimento, pessoal (equipe) e equipamento utilizado**

1. Data do procedimento
2. Tipo de procedimento
3. Nome do perfusionista(s)
4. Nome do cirurgião(ões)
5. Nome do anestesista(s)
6. Nome da enfermeira(s)
7. Número da sala de cirurgia
8. Comentários/eventos (recomendado)
9. Equipamento:
  - Máquina de CEC
  - *Cell saver* (autotransfusão)
  - Trocador de calor (*heater-cooler*)
  **Nota:** os itens a-c são geralmente identificados (p. ex., Máquina (Bomba) 1, 2, 3 etc.). Os números de série de cada um dos componentes (bomba de rolete, vaporizadores, *blender* etc.) devem estar documentados e arquivados em local apropriado.
10. Descartáveis:
  - Oxigenador
  - Reservatório de cardiotomia
  - Circuito de tubos e filtro arterial
  - Bomba centrífuga
  - Sistema de cardioplegia
  - Circuito de autotransfusão (*cell saver*)
  - Hemofiltro
  - Cânulas arteriais
  - Cânulas venosas
  - Cânulas de cardioplegia
  - Aspiradores e vent(s)
  **Nota:** o fabricante, modelo, número de série e/ou número do lote devem ser documentados em cada um dos itens (a-k) utilizados.

## Appendix C (Portuguese version)

**Parâmetros fisiológicos do paciente e ações do perfusionista documentadas com frequência definida pelo protocolo Institucional**

1. Fluxo sanguíneo de perfusão (RPM)
2. Pressão arterial
3. Pressão da linha arterial
4. Pressão venosa central ou pressão da artéria pulmonar
5. Retorno venoso assistido com vácuo (VAVR):
  - Pressão do reservatório venoso/da linha venosa
  - Pressão negativa do dispositivo de vácuo
6. Gasometria arterial e venosa
7. Saturação venosa de oxigênio
8. Temperaturas do paciente, incluindo:
  - Temperatura central (ao menos)
    - Nasofaríngea
    - Vesical
    - Esofágica
    - Retal e/ou
    - Timpânica
    - Temperatura do miocárdio (opcional)
9. Temperaturas da CEC:
  - Linha de retorno venoso
  - Linha arterial
  - Da água do trocador de calor (opcional)
10. Gases do oxigenador, incluindo fluxo de gases e concentração
11. Entrada de líquidos (volume infundido), incluindo:
  - Prime
  - Hemoderivados
  - Cristaloides
  - Solução cardioplégica
  - Componentes autólogos
12. Cardioplegia:
  - Solução (proporção se diferentes)
  - Via de administração (anterógrada, retrógrada etc.)
  - Fluxo
  - Pressão
  - Temperatura
  - Volume
13. Volume das perdas, incluindo:
  - Débito uninário
  - Ultrafiltrado
14. Medicações e/ou anestésicos inalatórios administrados através do circuito extracorpóreo

## Appendix D (Portuguese version)

**Resultados dos gases sanguíneos, eletrólitos e monitoração da anticogulação**

1. Gasometria:
  - pO_2_
  - pCO_2_
  - pH
  - Excesso de bases (BE)
  - Bicarbonato
  - Saturação
  - Concentração de potássio
  - Concentração de cálcio ionizado
  - Concentração de sódio
  - Lactato
  - Glicemia
  - Hemoglobina/hematócrito
2. Tempo de coagulação ativado (TCA) e/ou resultados da concentração plasmática de heparina/protamina e/ou resultado do tromboelastograma.

## Appendix E (Portuguese version)

**Avaliação pós-operatória dos pacientes submetidos à circulação extracorpórea**

1. Nome do paciente
2. Número hospitalar do paciente
3. Data da cirurgia
4. Hora de entrada na UTI
5. Valores de CK, CKMB e troponina de 6, 12 e 24h
6. Fração de ejeção no pós-operatório
7. Valores das drogas vasoativas utilizadas com 6, 12 e 24h
8. Balanço hídrico a cada 6h, até completar 48h de UTI
9. Pressão venosa central a cada 6h, até completar 48h de UTI
10. Uso de hemoderivados
11. Tempo de extubação
12. Data de alta da UTI

## Appendix F (Portuguese version)

**Checklist**

O *checklist* deve conferir (ao menos):

- Integridade e funcionamento da máquina de CEC e trocador de calor.
- Funcionamento dos sistemas de gases e as conexões com o oxigenador.
- Identificação correta do paciente.
- Disponibilidade de hemoderivados.
- Composição do perfusato.
- Composição e preparo da cardioplegia.
- Cânulas que serão utilizadas.
- Todas as conexões do circuito.
- Calibração e direção dos roletes.
- Calibração dos sensores e alarmes de pressão, fluxo e bolhas.
- Calibração dos sensores de pressão de cardioplegia.
- Calibração e conexão do monitor de gases.
- Disponibilidade de materiais descartáveis e equipamentos de emergência (hand crank, luz de emergência etc.)
- Dose e administração de heparina.
- Resultado de TCA antes de iniciar a CEC.
- Disponibilidade de fármacos, agulhas, seringas e equipos de soro e sangue para utilização durante a CEC.
